# Purification, Characterization, and Antioxidant Activity of Polysaccharides Isolated from Cortex Periplocae

**DOI:** 10.3390/molecules22111866

**Published:** 2017-10-31

**Authors:** Xiaoli Wang, Yifei Zhang, Zhikai Liu, Mingqin Zhao, Pengfei Liu

**Affiliations:** College of Tobacco Science/National Tobacco Cultivation & Physiology & Biochemistry Research Center, Henan Agricultural University, Zhengzhou 450002, China; xiaoliwang325@126.com (X.W.); zhangyifeiyc@126.com (Y.Z.); liuzhikaiyc@126.com (Z.L.)

**Keywords:** Cortex Periplocae, polysaccharides, purification, chemical composition, antioxidant activity

## Abstract

In this study, crude Cortex Periplocae polysaccharides (CCPPs) were extracted with water. CCPPs were decolored with AB-8 resin and deproteinated using papain-Sevage methods. Then, they were further purified and separated through DEAE-52 anion exchange chromatography and Sephadex G-100 gel filtration chromatography, respectively. Three main fractions—CPP1, CPP2, and CPP3, (CPPs)—were obtained. The average molecular weights, monosaccharide analysis, surface morphology, and chemical compositions of the CPPs were investigated by high-performance gel permeation chromatography (HPGPC), gas chromatography-mass spectrometry (GC/MS), UV-vis spectroscopy, Fourier transform infrared (FT-IR) spectrum, and nuclear magnetic resonance (NMR). In addition, the antioxidant activities of these three polysaccharides were investigated. The results indicated that all of the CPPs were composed of rhamnose, arabinose, mannose, glucose, and galactose. These three polysaccharides exhibited antioxidant activities in four assays including 1,1-diphenyl-2-picrylhydrazyl (DPPH) radical, 2,2′-azino-bis(3-ethyl-benzthiazoline-6-sulfonic acid) (ABTS) radical, reducing power, and total antioxidant activity in vitro. The data indicated that these three polysaccharides could be utilized as potential natural sources of alternative additives in the functional food, cosmetics, and pharmaceutical industries.

## 1. Introduction

In recent years, due to the development of new lifestyles and work pressure, there has been an increasing demand for functional foods, such as polyphenols, phospholipids, chitins, etc. To date, polysaccharides have been widely studied and found to be one of the most important biological macromolecules in Nature because of their wide range of pharmacological activities, such as antioxidant, antityrosinase, antitumor, antihypertensive, immune-enhancement, and many others [1,2,3,4,5,6,7]. As far as we know, at least thirty kinds of polysaccharides have been used around the world in clinical trials including anti-tumor, anti-virus, and diabetes therapy [8]. In China, polysaccharides have been developed into medicines, for example, Maitake Polysaccharides Capsule, Astragalus Polysaccharide Injection, Lentinan Injection, and Polysaccharides of G. *Lucidum* karst Injection. In addition, polysaccharides also have good water-holding capacity, fat-binding ability, emulsifying property, thermal stability, and sustained-release property, so they can be used in cosmetics, pharmaceuticals, food, and other products in various fields [7,9,10,11,12,13]. Therefore, more and more researchers are increasingly interested in discovering new polysaccharides from various sources. To the best of our knowledge, however, there are few published studies on the purification, chemical composition, or antioxidant activity of polysaccharides in Cortex Periplocae.

Cortex Periplocae, the dry root bark of *Periploca sepium* Bge, a perennial liana plant from the Asclepiadaceae family, grows widely in Shanxi, Shandong, Henan, and Hebei provinces in China. It is a commonly used Chinese herbal medicine due to its water swelling, dampness-dispelling, and bone-strengthening effects. Modern research has found that the water-soluble and fat-soluble extracts from Cortex Periplocae, such as baohuosidai-I, steroids, and triterpenes, possess analgesic and antitumor functions [14,15,16,17]. However, there has been no published literature on the extraction or purification of Cortex Periplocae polysaccharides or their structural characteristics and antioxidant capacities. Therefore, in the present study, the polysaccharides of Cortex Periplocae were extracted with water, decolored with AB-8 resin and deproteinized with papain-Sevage methods. The CCPPs were further purified with DEAE-52 and Sephadex G-100 chromatography, and three polysaccharides CPP1, CPP2, and CPP3 were obtained. The physical and chemical characteristics of the CPPs, such as Mw and monosaccharide composition, were determined. The structural features based on FT-IR, and NMR were determined. Furthermore, the antioxidant scavenging effects of the CPPs were evaluated using antioxidant assays, including DPPH radical, ABTS radical, reducing power, and total antioxidant activity in vitro.

## 2. Results and Discussion

### 2.1. Extraction, Isolation, and Purification of Polysaccharides

**Figure 1:**
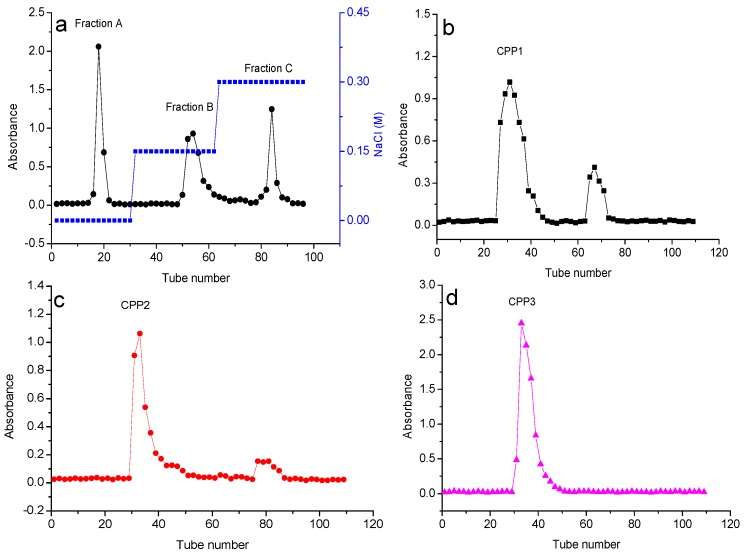
Elution curves of the CCPPs from the DEAE-52 column with NaCl solution (0, 0.15, and 0.3 M) (**a**); Elution curves of fractions A, B, and C from the Sephadex G-100 gel column with ultrapure water (**b**–**d**, respectively).

Crude polysaccharides were extracted with water. The yield was 3.7% by dry weight. Researchers commonly used anion exchange chromatography and size exclusion chromatography together to purify polysaccharides. The crude polysaccharides were decolorized with AB-8 resin and deproteinized with papain-sevage, successively. CCPPs were purified with the DEAE-52 anion-exchange column and three fractions, the water fraction (fraction A), 0.15 M NaCl fraction (fraction B), and 0.3 M NaCl fraction (fraction C; see Figure 1a), were obtained.

**Figure 2:**
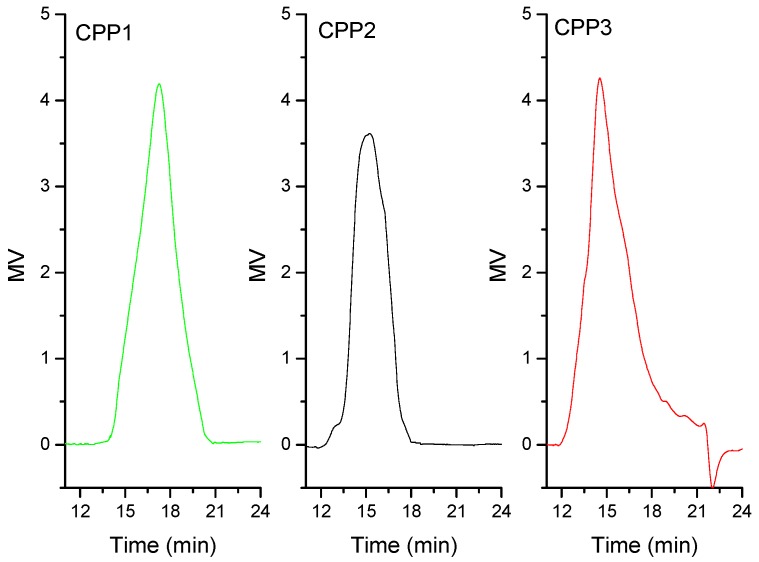
HPGPC chromatograms of CPP1, CPP2, and CPP3.

DEAE-52 is a negative anion exchange material, so according to the order of elution of these three fractions, the polarity of fraction A, B and C is increasing in that order. The three fractions were further purified with Sephadex G-100 eluted with water, and three subfractions were obtained: CPP1, CPP2, and CPP3 (Figure 1b–d). HPGPC equipped with refractive index detector is considered a powerful, effective, and reliable technique to determine the purity and molecular properties of polysaccharides. The HPGPC elution profiles of CPP1, CPP2, and CPP3 had single and symmetrically sharp peaks (Figure 2), which indicated the polysaccharides were homogeneous [18,19,20]. Based on the published literature, single and symmetrically sharp peaks can be obtained through HPGPC analysis [19,20], though some of the peaks were not perfect [18,21]. Researchers have accepted the results because the polysaccharides were mixtures of various degrees of polymerization, which made them difficult to obtain very homogeneous components. Based on the lower outputs measured for other elution fractions, CPP1, CPP2, and CPP3 were deemed to be the major polysaccharides in Cortex Periplocae for further research. The elution times of CPP1, CPP2, and CPP3 were 17.2, 14.5 and 15.2 min, and the average molecular weights of CPPs were 12.2, 34.4 and 15.9 kDa, respectively.

### 2.2. Analysis of Physicochemical and Monosaccharide Compositions

Table 1 shows the contents of the major chemical components of the three polysaccharides. The carbohydrate contents in CPP1, CPP2, and CPP3 were 75.23%, 82.44%, and 63.28%, respectively. Their protein and uronic acid levels were trace or undetectable.

**Figure 3:**
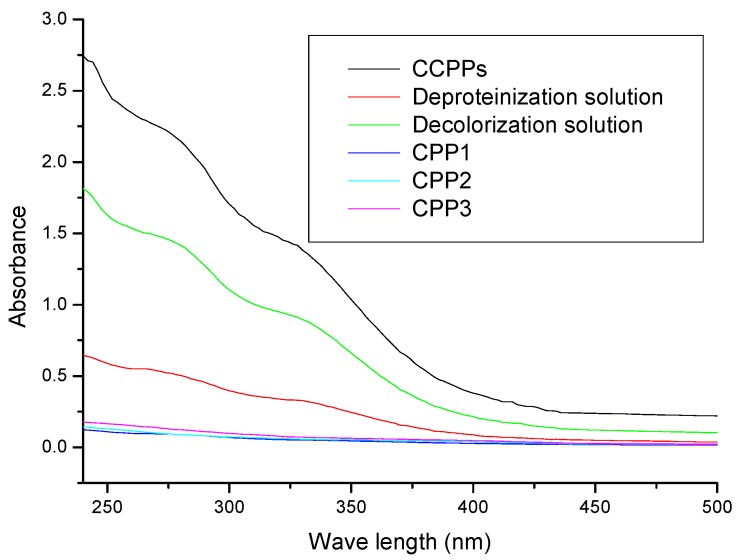
The UV-Vis spectra of the samples.

In the UV-Vis spectra, the absorption peak at 260 or 280 nm indicated that the samples might contain nucleic acids, proteins, or peptides [22,23,24]. As depicted in Figure 3, the CCPPs had a shoulder peak at 280 nm based on the UV-Vis spectra. The absorption of the decolorization solution at 280 nm was weaker than that of the CCPPs’, which showed that AB-8 resin also absorbed protein while adsorbing pigments. After the deproteinization treatment via the papain-Sevage method, the absorption peak at 280 nm became weaker, and the scanning curve showed a decreasing trend. However, there was almost no absorption peak at 260 or 280 nm based on the UV-vis spectra of CPP1, CPP2, and CPP3. The results were basically consistent with those of the chemical composition analysis.

**Figure 4:**
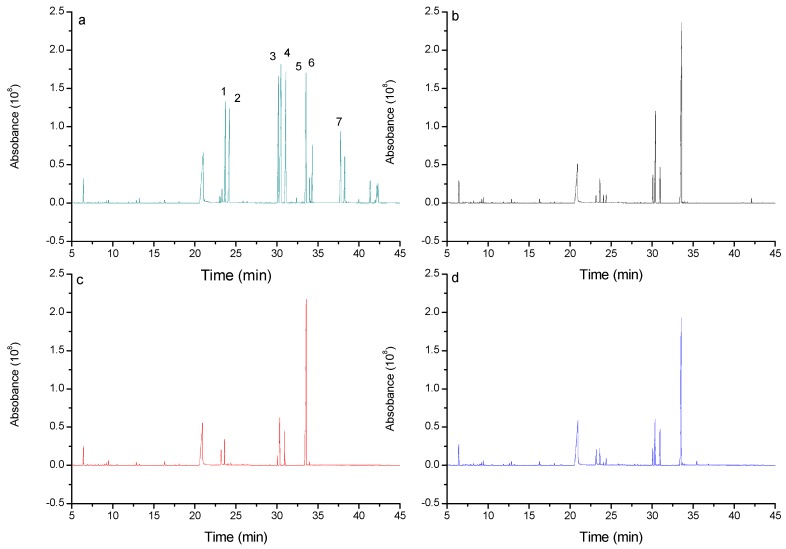
GC/MS profile of aldononitrile acetate derivatives of CPP1 (**b**), CPP2 (**c**), CPP3 (**d**), and monosaccharide standards ((**a**); 1, Rha; 2, Ara; 3, Man; 4, Glc; 5, Gal; 6, internal standard; and 7, Fru).

Figure 4 shows the GC/MS trace of the aldononitrile acetate derivatives of CPP1, CPP2, CPP3, and monosaccharide standards for comparison.

The CPPs determined by the GC/MS of the polysaccharides derivatives were composed of Rha, Ara, Man, Glc, and Gal. Table 1 depicts the molar ratios of the monosaccharides (Rha, Ara, Man, Glc, and Gal) as about 3%, 3%, 3%, 76%, and 15% in CPP1; 9%, 15%, 4%, 17%, and 55% in CPP2; and 16%, 3%, 3%, 44%, and 34% in CPP3. Based on the results, the monosaccharide composition of the three polysaccharides was similar, but the ratios of each monosaccharide varied greatly. CPP1 was rich in Glc and Gal; CPP2 was rich in Rha, Ara, Glc, and Gal; and CPP3 was rich in Rha, Glc, and Gal.

### 2.3. FT-IR Spectroscopic Analysis

**Figure 5:**
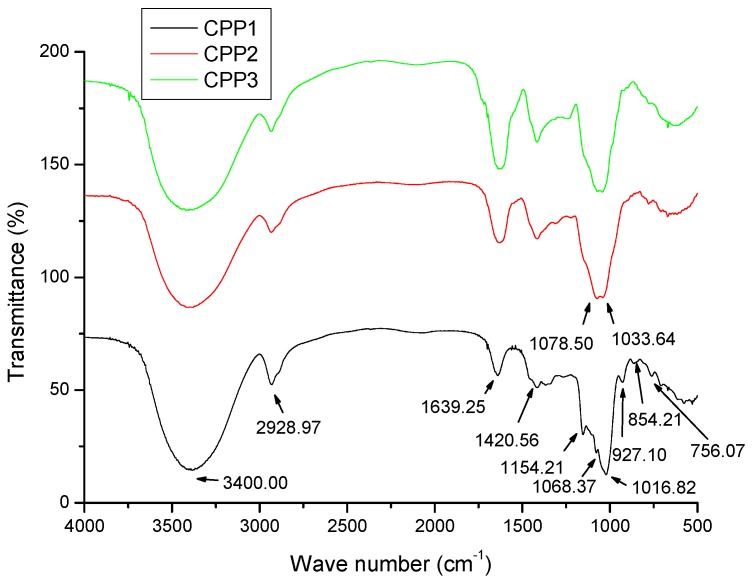
FT-IR spectra of CPP1, CPP2, and CPP3.

All of CPPs have similar FT-IR absorption bands, indicating similarities in their structural features. Figure 5 depicts the FT-IR spectra of CPPs in the 400–4000 cm^−1^ region. As described in the previous literatures, the broad, strongly represented intense band at 3400 cm^−1^ is due to the stretching vibration of O–H bonds [25,26]. The signal at around 2928 cm^−1^ can be associated with the stretching vibration of the C–H bond in the sugar ring [7]. The relative strong absorption peak at 1540–1650 cm^−1^ represents the characteristic stretching vibration of the C–O bond [27]. These three polysaccharides did not have the absorption peaks at 1541 cm^−1^, indicating that they did not contain protein [27], which was consistent with the Coomassie Brilliant Blue and UV-Vis spectra results. The band around 1420 cm^−1^ was assigned to the bending vibration of the C–H bond [28]. The polysaccharide peaks between 1000–1200 cm^−1^ were assigned to their C–O–C and C–O–H linkages [29]. The strong absorption band around 1050 cm^−1^ was assigned to the skeletal modes of pyranose rings in the monosaccharide of CPPs. The results showed that CPPs had strong absorption peaks of 1078.50, 1068.37, 1033.64 and 1016.82 cm^−1^, respectively, indicating that CPPs were rich in Gal, and Glc [30]. These results were consistent with the monosaccharide composition analyses. Carbohydrates have two conformers—the α- and β-conformers—which depend on the types of end carbonyl glycosidic bonds and might be discriminated based on the anomeric region-vibrational bands in the range of 750–950 cm^−1^ [31,32]. The characteristic absorption peaks around 854 and 756 cm^−1^ suggested that CPPs had α- and β- type glycosidic linkages between the sugars unites [33]. No absorption peak was detected at 1740 cm^−1^, which indicated there was no uronic acid in CPPs [29]. The results were consistent with the chemical composition analyses.

### 2.4. NMR Analysis

**Figure 6:**
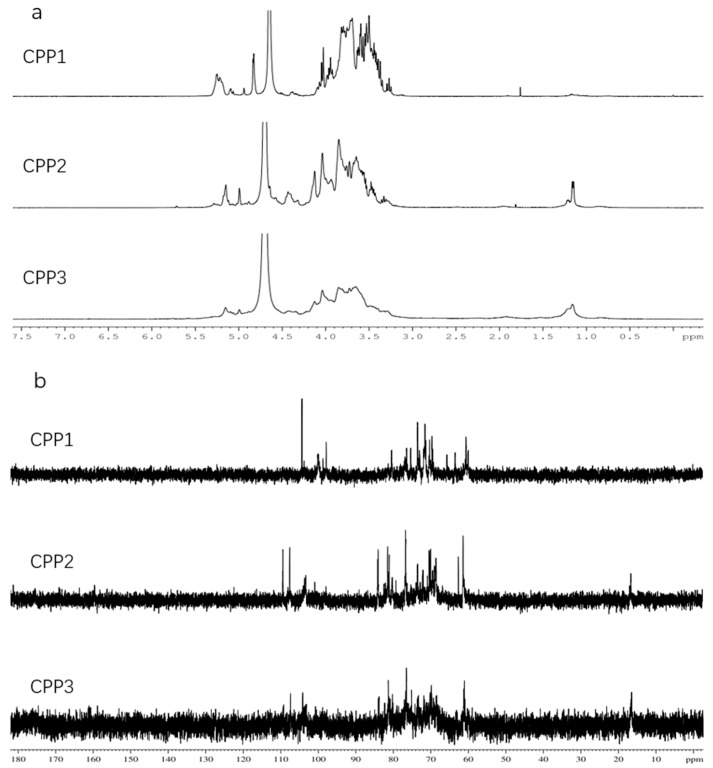
^1^H-NMR (**a**) and ^13^C-NMR (**b**) spectra of CPPs.

The structural features of the three polysaccharides were further elucidated through NMR spectral analysis. The 400-MHz ^1^H and ^13^C-NMR spectra of the CPPs are shown in Figure 6. The ^1^H-NMR results were consistent with the chemical composition analysis. The chemical shifts at 4.9–5.6 ppm and 4.3–4.9 ppm in the ^1^H-NMR spectrum could be assigned to the typical signals of the anomeric protons of α- and β-anomers, respectively [31]. Moreover, the anomeric protons at 4.9–5.5 ppm indicated that CPPs were mainly composed of several types of sugars [34]. The analysis showed that each of the three polysaccharides consisted of different monosaccharides, which meant that they were heteropolysaccharides. The peak at 4.7 ppm was deuterated water. Weak peaks of the samples around 4.4 ppm showed that these three polysaccharides contained a small amount of β-glycosidic bonds. Terminal methyl groups in compounds was showed ^1^H signals around 1.20 ppm [35]. In the ^13^C-NMR spectra, 90–110 ppm was the anomeric carbon area [34]. Each of the three polysaccharides contained several types of anomeric carbon signals, which indicated all of them were composed of a variety of sugars. The signal peak at 98.57 ppm was confirmed to be the anomeric carbon of α-d-Glc*p* [34,36]. In the ^13^C-NMR, the chemical shifts between 107.3–109.3 ppm were indicated to be Ara*f*; the chemical shifts between 102.9–104.2 ppm were indicated to be Gal*p* and Glc*p* [35,37]. The ^13^C signal from 67 to 70 ppm indicated the existence of (1 → 6) glycosidic linkages; the signals from 80 to 83 ppm confirmed the existence of (1 → 3/4) glycosidic linkages [35]. The presence of ^13^C-NMR signals at 170–180 ppm indicate the acetamino and carboxyl groups [34,38,39], while there were no corresponding signals in the ^13^C spectra of CPPs, which was consistent with the chemical composition analysis and FT-IR results.

### 2.5. Antioxidant Activity Assay

**Figure 7:**
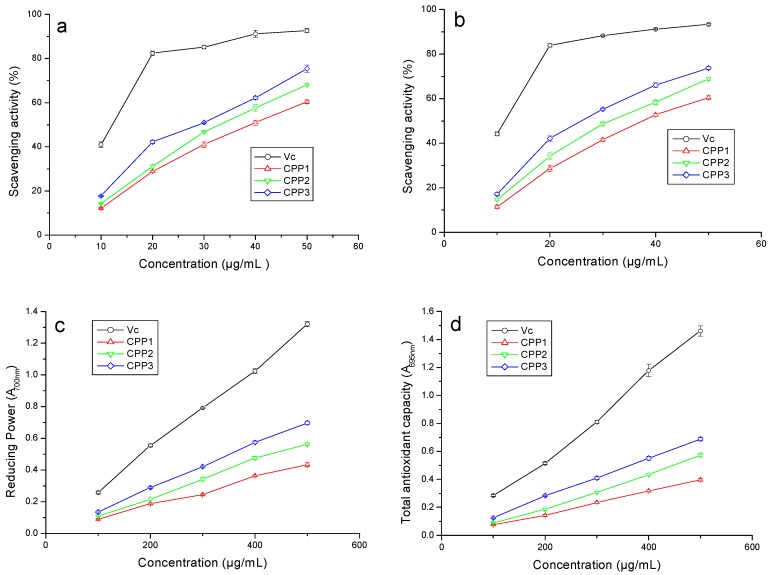
Antioxidant activities of CPP1, CPP2, and CPP3 scavenging of DPPH radical (**a**); scavenging of ABTS radical (**b**); reducing power (**c**); and total antioxidant activity (**d**).

DPPH is a stable free radical which shows maximum absorbance at 517 nm and is commonly used to measure the capacity of free-radical scavenging activities or hydrogen donation of food, cosmetics, and pharmacy materials [40]. As shown in Figure 7a, the rates of DPPH scavenging of these three polysaccharides increased fast with increasing concentration from 10 μg/mL to 50 μg/mL, indicating that the CPPs had significant scavenging activity on DPPH radicals. IC_50_ values of Vc, CPP1, CPP2, and CPP3 were 12.15, 39.20, 33.03, and 29.02 μg/mL, respectively. These results suggested that the scavenging activity of CPPs were not as good as the reference compound vitamin C (Vc). The ability of polysaccharides to release electrons or hydrogen to free radicals to terminate the free radical chain reaction is a possible mechanism [41].

The ABTS assay is a decolorizing method that is practicable for lipid-soluble and water-soluble antioxidant capacity and is commonly used in natural products, such as polysaccharides, polyphenols, and plasma [42]. As shown in Figure 7b, the CPPs presented significant effects on the scavenging ABTS radical ability, and the radical scavenging and ferric reducing abilities of CPP1, CPP2, and CPP3 were concentration-dependent. The IC_50_ values of Vc, CPP1, CPP2, and CPP3 were 11.48, 37.43, 31.47, and 25.84 μg/mL, respectively. As illustrated in Figure 7b, the ABTS scavenging ability order of the CPPs was the same as in the DPPH assay.

Reducing power activity measures the reductive ability, which was evaluated using the transformation of [Fe(CN)_6_]^3−^ to [Fe(CN)_6_]^4−^ in the presence of polysaccharides by donating an electron. Hence, the formation of Fe(II) can then be measured by the formation of Perl’s Prussian Blue at 700 nm; the higher absorbance values indicated higher ferric iron reducing power activity [43]. According to Figure 7c, the reductive capability results of the CPPs and Vc behaved in a concentration-dependent manner. Figure 7c shows that the reducing power activity of Vc increased quickly at concentrations from 100 to 500 μg/mL, and the reducing power activity of CPPs increased gradually with the increasing concentration. Nevertheless, the reducing power of the CPPs was weaker than that of Vc, which plays the role of the standard. Moreover, the order of scavenging ability was CPP3 > CPP2 > CPP1. At 500 μg/mL, the reducing capacities of CPP1, CPP2, and CPP3 were 0.43, 0.56, and 0.70, respectively. The reducing activity was generally related to react certain precursor of peroxides and to prevent peroxide formation [43]. Based on the theory, CPPs could have participated in the free radical reaction, provided electron donors, and converted them into more stable compounds.

The total antioxidant activity assay is usually performed to determine the antioxidant capacities of natural products such as polyphenols and polysaccharides. It was account of the meterage of TPTZ-Fe(II) complex generated by the reduction of the TPTZ-Fe(III) complex by polysaccharides [44]. A higher absorbance values indicated a stronger ferric reducing power activity of samples. In Figure 7d, the reducing power of CPP1, CPP2, CPP3 and reference standard Vc increased significantly with the increase of sample concentrations. The results showed that the reference standard presents an outstanding ability, higher than those of the CPPs. At 500 μg/mL, the reducing capacities of CPP1, CPP2, and CPP3 were 0.40, 0.57, and 0.69, respectively.

Overall, the antioxidant activity order of the CPPs in the present study was CPP3 > CPP2 > CPP1. Based on the published literatures, the antioxidant activity of natural polysaccharides might be related to their composition, molecular weight, water solubility, monosaccharide component, structure of chain conformation, polarity, and intramolecular hydrogen bonds [43,45]. Therefore, the antioxidant activity of polysaccharides is not a single factor but a combination of many factors. Some researchers have reported that polysaccharides with lower molecular weights present stronger reducing power [5,45], but Huang et al. reported that polysaccharides with larger molecular weights possessed higher reducing power [46]. In the present work, the results indicated that there was no obvious relationship between molecular weight and antioxidant activity, which was also consistent with reported results [47,48]. Uronic acids are usually considered as a key factor in antioxidant activity. Based on the reported literature, polysaccharides with a higher content of uronic acids possessed stronger antioxidant activities [2,33]. Wang et al. found that the antioxidant activity of neutral polysaccharides was significantly better than that of acidic polysaccharides [39]. Furthermore, some researchers have reported that polysaccharides with more Man and Rha displayed higher antioxidant activity [33,49]. In the present work, the higher combined contents of Man and Rha (CPP3 19%, CPP2 13%, and CPP1 6%, respectively) showed stronger antioxidant activity, which was consistent with the previous literature [33,49].

## 3. Materials and Methods

### 3.1. Materials

Cortex Periplocae produced in Shanxi Province of China was purchased from Zhang Zhongjing Pharmaceutical Ltd. (Zhengzhou, China) DEAE-52; Sephadex G-100; DPPH; ABTS; TCA; Vc (>99.7%); K_3_Fe(CN)_6_; papain; TFA; TPTZ; monosaccharide standards (Rha, Ara, Man, Glc, Gal, Fru, and GlcA); and Dextran Mw standards (T-10, T-40, T-100, T-500, and T-1000) were purchased from Beijing Solarbio Science & Technology Co., Ltd. (Beijing, China).

### 3.2. Extraction of Crude Polysaccharides

Dried Cortex Periplocae was milled into a powder and passed through a 40-mesh sieve. One kilogram of powder was extracted successively by petroleum ether and absolute ethanol at a ratio of 3:1 (*v*/*w*) for 4 h to remove the lipid-soluble and alcohol-soluble components. After filtration, the residue was dried, then extracted three times using ultrapure water at a ratio of 1:8 (*w*/*v*) for 3 h. After centrifugation (8000 rpm for 6 min), the supernatant was concentrated via rotary evaporator, and absolute ethyl alcohol was added into the concentrate to 80% to obtain deposition at 4 °C overnight. The deposition was washed with absolute ethanol and acetone, then freeze-dried using a vacuum freeze dryer (LYOVAC GT 2, SRK, Dusseldorf, Nordrhein-Westfalen, Germany).

### 3.3. Decolorization and Deproteinization

Before purification, the extract contained many impurities, mainly proteins and pigments. Ten grams of polysaccharide mixture was dissolved into 2 L ultrapure water; then, AB-8 resin was added and the solution was adjusted to a pH of 4 with hydrochloric acid and kept for 2 h. After that, the decolorizing solution was filtered. The filtrate was adjusted to a pH of 7 with a sodium hydroxide solution, followed by 0.1 g papain, and kept for 2 h to remove the protein. Later, the solution was subsequently mixed with Sevage reagent (butanol/chloroform, *v*/*v* = 1:4) for three times to remove the proteins. After the Sevage treatment, four volumes of ethanol were added to the aqueous solution at 4 °C overnight to obtain polysaccharides, and the precipitate was dissolved in ultrapure water. Then, the mixture was further completely dialyzed with ultrapure water for 72 h (Mw cutoff was 3.5 kDa) to remove the small molecules such as monosaccharides. Finally, the solution was lyophilized to obtain the CCPPs.

### 3.4. Purification of Polysaccharides

The CCPPs were purified using a previously described procedure with some modifications [35,47]. The CCPPs were dissolved in ultrapure water and centrifuged at 8000 rpm for 5 min. The supernatant was further purified and isolated with DEAE-52 anion exchange and Sephadex G-100 gel filtration chromatography. First, a DEAE-52 column (26 mm × 600 mm) was equilibrated with ultrapure water, followed by the elution of ultrapure water and sodium chloride solution (0.15 and 0.3 M) at a flow rate of 2.0 mL/min, successively. The elution was collected at 10 mL/tube, and the total carbohydrates were analyzed using the phenol-sulfuric acid method [50]. Based on the results, three main fractions (fraction A, B, and C) were obtained; then, these fractions were further completely dialyzed with ultrapure water for 72 h (Mw cutoff was 3.5 kDa). Later, the fractions were further fractionated with a Sephadex G-100 column (26 mm × 600 mm) and eluted with ultrapure water; the eluent was gathered at 6 mL/tube of eluent. Phenol-sulfuric acid method [50] was employed to estimate the total carbohydrate contents of the eluent. As a result, three purified fractions, CPP1, CPP2, and CPP3, were collected and lyophilized for subsequent analysis.

### 3.5. Characterization of the Purified Fractions

#### 3.5.1. Analysis of Chemical Characterization

Total carbohydrate contents of the CPPs were estimated by phenol-sulfuric acid method with Glc as the standard [50]. Uronic acid contents were estimated using a modified sulfuric acid-carbazole method with GlcA as the standard [51]. Protein contents were estimated through Coomassie Brilliant Blue method [52].

#### 3.5.2. Determination of Mw Distribution

The average molecule weight of CPPs was determined using HPGPC. Ten milligrams of CPPs were dissolved in 20 mL of ultrapure water to determine the Mw distribution and homogeneity of the purified polysaccharides. HPGPC was performed at 35 °C with an HPLC system (Waters 2695/2414, Waters, Milford, MA, USA) equipped with an Ultrahydrogel column (300 mm × 7.8 mm × 2 mm, exclusion limit 1 × 10^6^, Waters) and a differential refractive index detector. Dextran (T-10, T-40, T-100, T-500, and T-1000) were used as standards. The mobile phase was ultrapure water containing 0.1% sodium nitrate at a flow rate of 1.0 mL/min, and the injection volume was 20 μL with a concentration of 0.5 mg/mL. The average molecule weight values of the CPPs were estimated using standard dextran of known Mw. Then, the retention time vs. the logarithm of Mw of standard were used as the calibration curve to determine the Mw of the CPPs [21].

#### 3.5.3. Monosaccharide Composition Analysis

The monosaccharide composition of the CPPs was analyzed according to the reported literature method [53] with a few modifications. In brief, CPPs (10.0 mg) were hydrolyzed with trifluoroacetic acid (TFA, 8.0 mL 2.0 M) at 110 °C for 4 h. Then, the reaction was evaporated via rotary evaporation at 45 °C and the residue was distilled from three times with methanol to completely remove the TFA. The hydrolysate was evaporated and then mixed with hydroxylamine hydrochloride (10.0 mg), the internal standard inositol hexaacetyl ester (5.0 mg), and pyridine (0.50 mL) and then put into a 90 °C water bath to react for 30 min. After the reaction solution cooled to room temperature, acetic anhydride (0.50 mL) was added and the acetylation reaction was continued at 90 °C for 30 min. The derivatives were dried with N_2_, then extracted with dichloromethane and ultrapure water to acquire the pure acetate derivatives. Anhydrous sodium sulfate was used to remove the water in the organic phase, and the solution was filtered with a 0.45 μm nylon membrane. Then, 1.0 mL of the filtrate was analyzed by GC/MS (Agilent 7890B-5977A, Agilent, Wilmington, DE, USA) equipped with an HP-5MS capillary column (60 m × 0.25 mm × 0.25 μm). The standard monosaccharides (i.e., Rha, Ara, Man, Glc, Gal, and Fru) were analyzed by GC/MS in the same way as above. The column temperature increased from 130 °C (6 min) to 240 °C at 4 °C/min and held at 240 °C for 20 min. The injection temperature was 250 °C, and the flow rate of the He carrier gas was 1.0 mL/min. Quantification was performed from the peak areas using response factors from inositol six acetyl ester.

### 3.6. Spectrum Analysis

Ultraviolet/visible (UV-vis) spectrophotometric analysis was performed using the method of Zhang et al. [54] with some modifications. The absorbance of each sample solution was determined over the range of 240 to 500 nm using a UV-5200 scanning UV-vis spectrophotometer (Metash, Shanghai, China).

An FT-IR spectrophotometer (Nicolet iS50, Thermo Nicolet Co., Waltham, MA, USA) was used to determine the functional groups of the CPPs. Two milligrams of polysaccharides and 200 mg of spectroscopic-grade potassium bromide powder were pressed into a 1 mm wafer for FT-IR analysis in a wavenumber region of 400–4000 cm^−1^ and a resolution of 4 cm^−1^ [55].

CPPs were dissolved in D_2_O (supersaturated solution) to record ^1^H (64 scans) and ^13^C (15k scans) NMR spectra (with a BBFO-plus probe) on a Bruker spectrometer (Bruker, Rheinstetten, Germany) at 30 °C and operated at 400/100 MHz [34].

### 3.7. In Vitro Antioxidant Activity Test

#### 3.7.1. Scavenging Activity on DPPH Radicals Assay

DPPH radical scavenging capacity was measured as described by Seedevi et al. [45] with little modifications. In short, 1.5 mL of the CPPs and 2.0 mL of freshly prepared DPPH methanol solution (0.1 mM) were mixed together, then kept at 30 °C for 30 min in the darkness. The results of the reaction mixture were read at 517 nm with a UV-vis spectrophotometer. The scavenging activity on DPPH radicals was calculated with the equation as below:

(1) $$\mathrm{Scavenging}\;\mathrm{rate}(\%)=(1-\frac{A_{1}-A_{2}}{A_{0}})\times 100\%$$

where “A_0_” was the absorbance of blank control without any sample, “A_1_” was the absorbance of the reaction solution, and “A_2_” was the absorbance of solution without DPPH. Vc was used for comparison.

#### 3.7.2. ABTS Radical Scavenging Assay

The ABTS radical scavenging activity of the CPPs was carried out by referring to previous published literature with a few modifications [43,45]. Briefly, 7.4 mM ABTS solution was mixed with potassium persulfate in the darkness for 12 h. After that, the solution was diluted with 0.2 M sodium phosphate buffer (pH 7.4) to the absorbance of 0.70 ± 0.02 at 734 nm. One hundred milliliters of different concentrations of the CPPs water solution (5–50 μg/mL) and 2.9 mL of the ABTS solution were mixed well and reacted for 6 min. The reaction was immediately detected at 734 nm with a UV-vis spectrophotometer. The results were calculated using Equation (1), and the DPPH solution was instead determined by ABTS. Vc was used for comparison.

#### 3.7.3. Reducing Power Ability Assay

The reducing power was measured according to the literature [43] with some modifications. Briefly, 1.0 mL of different concentrations of the CPPs water solution (100–500 μg/mL), 2.5 mL potassium ferricyanide solution (1%, *w*/*v*), and 2.5-mL sodium phosphate buffer (0.2 M, pH 6.6) were added together. The reaction was mixed vigorously and placed at 50 °C for 20 min. After that, 2.5 mL TCA (10%, *w*/*v*) was put into the solution to end the reaction, and the mixed solution was centrifuged at 5000 rpm for 10 min. Then, 2.5 mL supernatant, 0.5 mL ferric chloride solution (0.1%, *w*/*v*), and 2.0 mL ultrapure water were mixed vigorously. The absorbance was read at 700 nm with a UV-vis spectrophotometer against a blank after 10 min. Vc was used for comparison.

#### 3.7.4. The Total Antioxidant Activity Assay

The total antioxidant activity was measured by referring to the method described in the literature [43], with some modifications. Briefly, 1.0 mL samples with different concentrations (100–500 μg/mL) were mixed with 3.0 mL reagent solution [0.6 M sulphuric acid, 28 mM sodium phosphate, and 4 mM ammonium molybdate]. The reaction mixture was placed in a water bath at 95 °C for 90 min. After the mixture cooled to room temperature, the absorbance of each mixture was read at 695 nm with a UV-vis spectrophotometer. The higher absorbance showed the higher total antioxidant activity. Vc was used for comparison.

### 3.8. Statistical Analysis

Each of the experiments was performed three times, and the data were presented as average ± standard deviation (SD). One-way analysis of variance was carried out to consider the significant difference (*p* < 0.05) by SPSS 19.0 (IBM SPSS, Chicago, IL, USA).

## 4. Conclusions

In conclusion, in the present study three novel polysaccharides (CPP1, CPP2, and CPP3) were successfully obtained from Cortex Periplocae. The monosaccharide composition detected by GC/MS indicated that the CPPs were heteropolysaccharides with different molar ratios of Rha, Ara, Man, Glc, and Gal. CPP1 was rich in Glc and Gal; CPP2 was rich in Rha, Ara, Glc, and Gal; and CPP3 was rich in Rha, Glc, and Gal. The average molecular weights of CPP1, CPP2, and CPP3 detected by HPGPC were 12.2, 34.4, and 15.9 kDa, respectively. From the FT-IR spectra, characteristic absorption peaks of polysaccharide were found and there was no absorption peak at 1740 cm^−1^. The results showed that there was no uronic acid in CPPs. In addition, according to the ^13^C-NMR results, there were no acetamino or carboxyl group signals at 170–180 ppm in the CPPs. Overall, CPPs are neutral heteropolysaccharides on the basis of sulfuric acid-carbazole, IR and NMR results. What’s more, all purified polysaccharides showed significant antioxidant activities in four in vitro assays. CPP3 showed stronger antioxidant activity, probably resulting from the larger amount of Man and Rha. The overall results indicated that CPPs could be utilized as potential natural antioxidants in the pharmacy, cosmetic, and food industries. Further studies are to investigate the relationship between the structure and the antioxidant activity would be desirable.

## Figures and Tables

**Table 1:** Major chemical composition of the CPPs.

Fragments	CPP1	CPP2	CPP3
Total carbohydrate (%)	75.23 ± 1.38	82.44 ± 2.57	63.28 ± 1.66
Protein (%)	-	-	-
Uronic acid (%)	-	-	-
Monosaccharide composition (%)			
Rha	3	9	16
Ara	3	15	3
Man	3	4	3
Glc	76	17	44
Gal	15	55	34

-, Trace or undetectable.

## Abbreviations

CCPPs	crude Cortex Periplocae polysaccharides
HPGPC	high-performance gel permeation chromatography
GC/MS	gas chromatography-mass spectrometry
FT-IR	Fourier transform infrared
NMR	nuclear magnetic resonance
Mw	molecular weight
DPPH	1,1-diphenyl-2-picrylhydrazyl
ABTS	2,2′-azino-bis (3-ethylbenzthiazoline-6-sulfonic acid)
Rha	d-rhamnose
Ara	d-arabinose
Man	d-mannose
Glc	d-glucose
Gal	d-galactose
Fru	d-fructose
GlcA	glucuronic acid
TPTZ	2,4,6-tripyridyl-*S*-triazine
TFA	trifluoroacetic acid
TCA	trichloroacetic acid

## References

[1] Wang J., Lu H.D., Muḥammad U., Han J.Z., Wei Z.H., Lu Z.X., Bie X.M., Lu F.X. (2016). Ultrasound-assisted extraction of polysaccharides from *Artemisia selengensis* Turcz and its antioxidant and anticancer activities nanobiotechnology. J. Food Sci. Technol..

[2] He P.F., Zhang A.Q., Zhang F.M., Linhardt R.J., Sun P.L. (2016). Structure and bioactivity of a polysaccharide containing uronic acid from *Polyporus umbellatus* sclerotia. Carbohyd. Polym..

[3] Ye D.Y., Jiang Z.B., Zheng F.C., Wang H.M., Zhang Y.M., Gao F.F., Chen P.H., Chen Y.C., Shi G. (2015). Optimized Extraction of Polysaccharides from *Grateloupia livida* (Harv.) Yamada and Biological Activities. Molecules.

[4] Wang L.B., Liu F.C., Wang A.X., Yu ZY., Xu Y.Q., Yang Y. (2017). Purification, characterization and bioactivity determination of a novel polysaccharide from pumpkin (*Cucurbita moschata*) seeds. Food Hydrocoll..

[5] Chen B.J., Shi M.J., Cui S., Hao S.X., Hider R.C., Zhou T. (2016). Improved antioxidant and anti-tyrosinase activity of polysaccharide from *Sargassum fusiforme* by degradation. Int. J. Biol. Macromol..

[6] Wu Y., Yi L., Li E.T., Li Y.Y., Lu Y., Wang P.J., Zhou H.L., Liu J.G., Hu Y.L., Wang D.Y. (2017). Optimization of *Glycyrrhiza polysaccharide* liposome by response surface methodology and its immune activities. Int. J. Biol. Macromol..

[7] Romdhanea M.B., Haddara A., Ghazala I., Jeddou K.B., Helbert C.B., Chaabouni S.E. (2017). Optimization of polysaccharides extraction from watermelon rinds: Structure, functional and biological activities. Food Chem..

[8] Shi L. (2016). Bioactivities, isolation and purification methods of polysaccharides from natural products: A review. Int. J. Biol. Macromol..

[9] Preethi S., Saral M. (2016). Screening of natural polysaccharides extracted from the fruits of *Pithecellobium dulce* as a pharmaceutical adjuvant. Int. J. Biol. Macromol..

[10] Zhu Z.Y., Liu F., Gao H., Sun H.Q., Meng M., Zhang Y.M. (2016). Synthesis, characterization and antioxidant activity of selenium polysaccharide from *Cordyceps militaris*. Int. J. Biol. Macromol..

[11] Ma F.Y., Zhang Y., Wen Y.R., Yao Y.N., Zhu J.H., Liu X.H., Bell A., Tikkanen-Kaukanen C. (2017). Emulsification properties of polysaccharides from *Dioscorea opposite* Thunb. Food Chem..

[12] Pang Z.H., Deeth H., Bansal N. (2015). Effect of polysaccharides with different ionic charge on the rheological, microstructural and textural properties of acid milk gels. Food Res. Int..

[13] Horvat G., Fajfar T., Uzunalic A.P., Knez Z., Novak Z. (2017). Thermal properties of polysaccharide aerogels. J. Therm. Anal. Calorim..

[14] Huang Y., Liu L.J., Li J., Bi Z.M. (2014). Study on antitumor activity and structure-activity relationship of C21 steroids from Periplocae Cortex. China J. Chin. Med..

[15] Wang L.F., Meng F.R., Zhou Y., Cao Q., Shan B.E. (2012). Effect of triterpenes compound of cortex periplocae on PCNA expression in ratesophageal carcinoma. China J. Cancer Biother..

[16] Zhu L.L., Bao Z.Y., Wang H., Huang W., Sun R. (2012). The analgesic effect study on margin of safety on different components from Cortex Periplocae. Chin. J. Pharm..

[17] Zhao L.M., Wang X.H., Yan X., Geng Y.M., Wang L., Liu L.H., Shan B.E. (2012). Mechanism of Baohuosidde-I from Cortex Periplocae inhibits cell proliferarion of human esophageal carcinoma. Cancer Res. Prev. Treat..

[18] Li C., Li X.S., You L.J., Fu X., Liu R.H. (2017). Fractionation, preliminary structural characterization and bioactivities of polysaccharides from *Sargassum pallidum*. Carbohyd. Polym..

[19] Chen J.L., Pang W.S., Shi W.T., Yang B., Kan Y.J., He Z.D., Hu J. (2016). Structural elucidation of a novel polysaccharide from *Pseudostellaria heterophylla* and Stimulating Glucose Uptake in cells and distributing in rats by oral. Molecules.

[20] Yin J.Y., Nie S.P., Zhou C., Wan Y., Xie M.Y. (2010). Chemical characteristics and antioxidant activities of polysaccharide purified from the seeds of *Plantago asiatica* L.. J. Sci. Food Agric..

[21] Cui C., Lu J.H., Sun-Waterhouse D.X., Mu L.X., Sun W.Z., Zhao M.M., Zhao H.F. (2016). Polysaccharides from *Laminaria japonica*: Structural characteristics and antioxidant activity. LWT-Food Sci. Technol..

[22] Shang X.Y., Chao Y., Zhang Y., Lu C.Y., Xu C.L., Niu W.N. (2016). Immunomodulatory and antioxidant effects of polysaccharides from *Gynostemma pentaphyllum* Makino in immunosuppressed mice. Molecules.

[23] Zou Y., Zhao T., Mao G.H., Zhang M., Zheng D.H., Feng W.W., Wang W.W., Wu X.Y., Yang L.Q. (2014). Isolation, purification and characterization of selenium-containing polysaccharides and proteins in selenium-enriched Radix puerariae. J. Sci. Food Agric..

[24] Hu T., Huang Q.L., Wong K.H., Yang H. (2017). Structure, molecular conformation, and immunomodulatory activity of four polysaccharide fractions from *Lignosus rhinocerotis* sclerotia. Int. J. Biol. Macromol..

[25] Chokboribal J., Tachaboonyakiat W., Sangvanich P., Ruangpornvisuti V., Jettanacheawchankit S., Thunyakitpisal P. (2015). Deacetylation affects the physical properties and bioactivity of acemannan, an extracted polysaccharide from *Aloe vera*. Carbohyd. Polym..

[26] Chawananorasest K., Saengtongdee P., Kaemchantuek P. (2016). Extraction and characterization of Tamarind (*Tamarind indica* L.) seed polysaccharides (TSP) from three difference sources. Molecules.

[27] Zhang F., Lin L.H., Xie J.H. (2016). A mini-review of chemical and biological properties of polysaccharides from *Momordica charantia*. Int. J. Biol. Macromol..

[28] Khatua S., Acharya K. (2016). Influence of extraction parameters on physico-chemical characters and antioxidant activity of water soluble polysaccharides from *Macrocybe gigantea* (Massee) Pegler & Lodge. J. Food Sci. Technol..

[29] Pu J.B., Xia B.H., Hu Y.J., Zhang H.J., Chen J., Zhou J., Liang W.Q., Xu P. (2015). Multi-optimization of ultrasonic-assisted enzymatic extraction of Atratylodes macrocephala polysaccharides and antioxidants using response surface methodology and desirability function approach. Molecules.

[30] Kacurakova M., Capek P., Sasinkova V., Wellner N., Ebringerova A. (2000). FT-IR study of plant cell wall model compounds: Pectic polysaccharides and hemicelluloses. Carbohyd. Polym..

[31] He S.D., Wang X., Zhang Y., Wang J., Sun H.J., Wang J.H., Cao X.D., Ye Y.K. (2016). Isolation and prebiotic activity of water-soluble polysaccharides fractions from the bamboo shoots (*Phyllostachys praecox*). Carbohyd. Polym..

[32] Wang L., Liu H.M., Xie A.J., Wang X.D., Zhu C.Y., Qin G.Y. (2017). Chinese quince (*Chaenomeles sinensis*) seed gum: Structural characterization. Food Hydrocoll..

[33] Zhang Q.H., Yu J.B., Zhang L.F., Hu M.Q., Xu Y., Su W.K. (2016). Extraction, characterization, and biological activity of polysaccharides from *Sophora flavescens* Ait. Int. J. Biol. Macromol..

[34] Wang X.T., Zhu Z.Y., Zhao L., Sun H.Q., Meng M., Zhang J.Y., Zhang Y.M. (2016). Structural characterization and inhibition on α-d-glucosidase activity of non-starch polysaccharides from *Fagopyrum tartaricum*. Carbohyd. Polym..

[35] Yu X.H., Liu Y., Wu X.L., Liu L.Z., Fu W., Song D.D. (2017). Isolation, purification, characterization and immunostimulatory activity of polysaccharides derived from American ginseng. Carbohyd. Polym..

[36] Popov S.V., Ovodova R.G., Golovchenko V.V., Popova G.Y., Viatyasev F.V., Shashkov A.S. (2011). Chemical composition and anti-inflammatory activity of a pectic polysaccharide isolated from sweet pepper using a simulated gastric medium. Food Chem..

[37] Zou Y.F., Fu Y.P., Chen X.F., Austarheim I., Inngjerdingen K.T., Huang C., Eticha L.D., Song X., Li L.X., Feng B. (2017). Purification and partial structural characterization of a complement fixating polysaccharide from Rhizomes of *Ligusticum chuanxiong*. Molecules.

[38] Kolsi R.B.A., Fakhfakh J., Krichen F., Jribi I., Chiarore A., Patti F.P., Blecker C., Allouche N., Belghith H., Belghith K. (2016). Structural characterization and functional properties of antihypertensive *Cymodocea nodosa* sulfated polysaccharide. Carbohyd. Polym..

[39] Wang L., Liu H.M., Qin G.Y. (2017). Structure characterization and antioxidant activity of polysaccharides from Chinese quince seed meal. Food Chem..

[40] Elnahas M.O., Amin M.A., Hussein M.M.D., Shanbhag V.C., Ali A.E., Wall J.D. (2017). Isolation, Characterization and Bioactivities of an Extracellular Polysaccharide Produced from Streptomyces sp. MOE6. Molecules.

[41] Bouaziz F., Koubaa M., Helbert C.B., Kallel F., Driss D., Kacem I., Ghorbel R., Chaabouni S.E. (2015). Purification, structural data and biological properties of polysaccharide from *Prunus amygdalus* gum. Int. J. Food Sci. Technol..

[42] Di T., Chen G.J., Sun Y., Ou S.Y., Zeng X.X., Ye H. (2017). Antioxidant and immunostimulating activities in vitro of sulfated polysaccharides isolated from *Gracilaria rubra*. J. Funct. Foods.

[43] Raza A., Li F., Xu X.Q., Tang J. (2017). Optimization of ultrasonic-assisted extraction of antioxidant polysaccharides from the stem of *Trapa quadrispinosa* using response surface methodology. Int. J. Biol. Macromol..

[44] Shi M.J., Wei X.Y., Xu J., Chen B.J., Zhao D.Y., Cui S., Zhou T. (2017). Carboxymethylated degraded polysaccharides from *Enteromorpha prolifera*: Preparation and in vitro antioxidant activity. Food Chem..

[45] Li F.W., Gao J., Xue F., Yu X.H., Shao T. (2016). Extraction optimization, purification and physicochemical properties of polysaccharides from *Gynura medica*. Molecules.

[46] Huang K.W., Li Y.R., Tao S.C., Wei G., Huang Y.C., Chen D.F., Wu C.F. (2016). Purification, characterization and biological activity of polysaccharides from *Dendrobium officinal*. Molecules.

[47] Zhou X., Wang H.D., Wang B.L., Fu L., Yuan M., Liu J., Zhou L.J., Ding C.B. (2016). Characterization and antioxidant activities of polysaccharides from the leaves of *Lilium lancifolium* Thunb. Int. J. Biol. Macromol..

[48] Zeng H.L., Miao S., Zheng B.D., Lin S., Jian Y.Y., Chen S., Zhang Y. (2015). Molecular structural characteristics of polysaccharide fractions from *Canarium album* (Lour.) Raeusch and their antioxidant activities. J. Food Sci..

[49] Thambiraj S.R., Phillips M., Koyyalamudi S.R., Reddy N. (2015). Antioxidant activities and characterisation of polysaccharides isolated from the seeds of *Lupinus angustifolius*. Ind. Crops Prod..

[50] Dubois M., Gilles K.A., Hamilton J.K., Rebers P., Smith F. (1956). Colorimetric method for determination of sugars and related substances. Anal. Chem..

[51] Blumenkrantz N., Asboe-Hansen G. (1973). New method for quantitative determination of uronic acids. Anal. Biochem..

[52] Bensadoun A., Weinstein D. (1976). Assay of proteins in the presence of interfering materials. Anal. Biochem..

[53] Sawardekar J.S., Slonekar L.S., Jeanes A. (1967). Quantitative determination of monosaccharides as their alditol acetates by gas liquid chromatography. Anal. Chem..

[54] Zhang W.J., Huang J., Wang W., Li Q., Chen Y., Feng W.W., Zheng D.H., Zhao T., Mao G.H., Yang L.Q. (2016). Extraction, purification, characterization and antioxidant activities of polysaccharides from *Cistanche tubulosa*. Int. J. Biological. Macromol..

[55] Liu H.Z., Jiang N., Liu L., Sheng X.J., Shi A.M., Hu H., Yang Y., Wang Q. (2016). Extraction, purification and primary characterization of polysaccharides from Defatted Peanut (*Arachis hypogaea*) Cakes. Molecules.

